# Cooperative Effects of Akt-1 and Raf-1 on the Induction of Cellular Senescence in Doxorubicin or Tamoxifen Treated Breast Cancer Cells

**DOI:** 10.18632/oncotarget.315

**Published:** 2011-08-30

**Authors:** Jackson R. Taylor, Brian D. Lehmann, William H. Chappell, Stephen L. Abrams, Linda S. Steelman, McCubrey McCubrey

**Affiliations:** ^1^Department of Microbiology & Immunology, Brody School of Medicine at East Carolina University Greenville, NC 27858 USA; ^2^Department of Anatomy & Cell Biology, Brody School of Medicine at East Carolina University, Greenville, NC 27858 USA; ^*^Wake Forest University, Winston-Salem, NC, USA; ^#^Vanderbilt University, Nashville, TN, USA

**Keywords:** Akt, ERK, mTOR, Senescence, Drug Resistance, Tamoxifen

## Abstract

Escape from cellular senescence induction is a potent mechanism for chemoresistance. Cellular senescence can be induced in breast cancer cell lines by the removal of estrogen signaling with tamoxifen or by the accumulation of DNA damage induced by the chemotherapeutic drug doxorubicin. Long term culturing of the hormone-sensitive breast cancer cell line MCF-7 in doxorubicin (MCF-7/DoxR) reduced the ability of doxorubicin, but not tamoxifen, to induce senescence. Two pathways that are often upregulated in chemo- and hormonal-resistance are the PI3K/PTEN/Akt/mTOR and Ras/Raf/MEK/ERK pathways. To determine if active Akt-1 and Raf-1 can influence drug-induced senescence, we stably introduced activated ΔAkt-1(CA) and ΔRaf-1(CA) into drug-sensitive and doxorubicin-resistant cells. Expression of a constitutively-active Raf-1 construct resulted in higher baseline senescence, indicating these cells possessed the ability to undergo oncogene-induced-senescence. Constitutive activation of the Akt pathway significantly decreased drug-induced senescence in response to doxorubicin but not tamoxifen in MCF-7 cells. However, constitutive Akt-1 activation in drug-resistant cells containing high levels of active ERK completely escaped cellular senescence induced by doxorubicin and tamoxifen. These results indicate that up regulation of the Ras/PI3K/PTEN/Akt/mTOR pathway in the presence of elevated Ras/Raf/MEK/ERK signaling together can contribute to drug-resistance by diminishing cell senescence in response to chemotherapy. Understanding how breast cancers containing certain oncogenic mutations escape cell senescence in response to chemotherapy and hormonal based therapies may provide insights into the design of more effective drug combinations for the treatment of breast cancer.

## INTRODUCTION

Signal transduction cascades downstream of epidermal growth factor (EGF) receptor (EGFR) isoforms (*e.g*., EGFR & HER2) have been associated with breast cancer development and resistance to anticancer agents [1]. Among the signaling pathways downstream of these receptors, the Ras/Raf/MEK/ERK and PI3K/PTEN/Akt/mTOR pathways have been shown to regulate apoptosis and their deregulation is often implicated in malignant transformation [2-9]. Indeed, the PI3K p110 catalytic subunit gene (*PIK3CA*) is one of the most frequently mutated genes in breast cancer [10-11]. Oncogenic mutations often elicit a permanent cell cycle withdrawal, termed oncogene-induced-senescence (OIS), to eliminate pre-malignant cells before they acquire additional mutations [12].

Phosphatidylinositol (PI) (3,4)P^2^ and PI(3,4,5)P^3^ are produced by class 1A PI3Ks and recruit phosphoinositide dependent kinase-1 (PDK1) as well as Akt isoforms to the plasma membrane by interacting with their pleckstrin homology (PH) domains [13-17]. Colocalization of PDK1 with Akt proteins at the plasma membrane causes PDK1 to phosphorylate Akt proteins at a threonine residue (T308) [18-19] and a serine residue (S473). Activation of PDK1 and Akt by class 1A PI3Ks is negatively regulated by phosphatase and tensin homologue deleted on chromosome ten (PTEN) [4,6,8,16,20,21]. PTEN removes phosphate groups from PI(3,4)P^2^ and PI(3,4,5)P^3^ added by PI3K as well as from tyrosine phosphorylated proteins including focal adhesion kinase (FAK) and Shc [4,16,20,22]. PTEN also has important roles in the nucleus and can regulate genome stability [6, 21]. In some cases, there are complex interactions with the p53, PI3K/PTEN/Akt/mTOR pathways which determine whether senescence, quiescence or autophagy occurs in response to DNA damaging drugs [12, 15, 23-49].

Diverse mechanisms regulate PTEN expression [7, 50-53], including gene deletion, alterations in mRNA splicing, subcellular localization, epigenetic repression and protein:protein interactions [51]. There are numerous microRNAs (miRNAs) which target the PTEN gene to inhibit its expression [4,6,50,54,55]. Furthermore there is a pseudo PTEN gene [6] which serves as a decoy to bind and neutralize some of these miRNAs. PTEN interacts with p53 to influence key points in the regulation of cellular proliferation. Loss of these tumor suppressor interactions can lead to cancer or in some cases cellular senescence [12,53,56].

PTEN mutations occur in breast cancer at varying frequencies (5-21%). While PTEN is deleted in certain cancers, loss of heterozygosity (LOH) is a more common genetic event (30%) leading to decreased PTEN expression [50,57]. Additionally, PTEN expression can be decreased by promoter methylation [50]. In one study, 26% of primary breast cancers had low PTEN levels which correlated with lymph node metastases and poor prognoses [58]. Disruption of PTEN activity by various mechanisms frequently results in Akt activation.

Elevated Akt activity can have pleiotropic effects on cell growth, which include activation/inactivation of transcription factors controlling pivotal gene expression, inactivation of pro-apoptotic molecules by their phosphorylation and subsequent proteasomal degradation, or by regulating the efficiency of translation of mRNAs involved in growth. Akt can regulate translation directly by activating mammalian target of rapamycin (mTOR) or indirectly by inhibiting TSC2. Active mTOR then can phosphorylate ribosomal S6 kinase (p70S6K), which in turn regulates the efficiency of translation of certain mRNAs and also functions in a negative feedback loop to control Akt activity [7,16,59-61]. The activities of Akt and p70S6K along with other molecules are essential for the formation of the eIF4F translation complex. The eIF4F complex is necessary for the translation of mRNAs containing long 5'UTRs which are highly-structured and have an elevated G+C content. These “weak” mRNAs often encode proteins involved in oncogenesis and survival such as c-Myc, Mcl-1, cyclin-D, VEGF and survivin. Furthermore p70S6K has important roles in autophagy and cellular senescence [62-65].

Akt, mTOR and p70S6K activation have been associated with a more severe prognosis in breast and other cancers [66-73]. Targeting the PI3K/PTEN/Akt/mTOR pathway may prove effective therapy in a variety of cancers [9,16,74-76]. Indeed some studies have evaluated the effectiveness of targeting mTOR in PTEN-negative cells [69]. Elevated active Akt expression has been associated with both chemo- and hormonal resistance in breast cancer [67-68,77]. Cells expressing activated Akt may be more sensitive to mTOR inhibitors such as rapamycin and may increase the efficacy in combination with chemo- and hormonal-based therapies [6,69]. A distinct advantage of targeting mTOR with rapamycin is that it has been used for many years to treat organ transplant patients. Rapamycin is now being examined in treatment of certain cancers and in the prevention of aging and other diseases including AIDS [7,8,78]. Previously it was determined that mutated forms of Akt and PTEN can induce chemotherapeutic- and hormonal-based drug resistance in breast cancer [5,67,77]. PTEN mutants that eliminate the lipid phosphatase activity result in activated Akt expression and drug resistance, which can be reversed by the mTOR inhibitor rapamycin [5].

In addition to deregulation of the PI3K/mTOR pathway, the Ras/Raf/MEK/ERK pathway plays a critical role in cellular transformation and drug resistance. Growth factor/cytokine/mitogen activation recruits a Src homology 2 (Shc) domain containing adaptor protein to the C-terminus of the receptor [6-8,60]. Shc in turn recruits the growth factor receptor-bound protein 2 (Grb2) protein and the son of sevenless (SOS) homolog protein, resulting in the loading of membrane-bound Ras with GTP [6-8,60]. Additionally, Ras can be activated by growth factor receptor tyrosine kinases (RTK), such as insulin receptor (IR), and insulin-like growth factor one receptor (IGF1-R), via intermediates like insulin receptor substrate (IRS) proteins that bind Grb2 [6-8,60,79]. Ras:GTP can recruit Raf to the membrane where it becomes activated, likely via a Src-family tyrosine kinase [6-8,60,80,81]. Ras and Raf are members of multi-gene families and there are three Ras members (*KRAS*, *NRAS* and *HRAS*) and three Raf members (*BRAF*, *CRAF* (*a.k.a* Raf-1) and *ARAF*) [6-8,60]. Raf is responsible for serine/threonine phosphorylation of mitogen-extracellular activated protein kinase kinase-1 (MEK1) [6-8,60]. MEK1 phosphorylates extracellular signal-regulated kinase 1 and 2 (ERK1/2) at specific threonine and tyrosine residues [6-8,60]. Activated ERK1 and ERK2 serine/threonine kinases phosphorylate and activate a variety of substrates, including p90 ribosomal six kinase (p90^Rsk1^) [6-8,60,82,83]. ERK1/2 has many downstream (>60) and even upstream substrates. The Ras/Raf/MEK/ERK pathway plays pivotal roles in: differentiation, chemotherapeutic drug resistance, cellular senescence and metastasis [2,84-94]. Thus suppression of MEK and ERK activities will have profound effects on cell growth. While Ras is frequently mutated in human cancers overall, it is rarely mutated in breast cancer. However, this pathway can be activated by multiple other mechanisms including: mutations in upstream receptors, mutations in pathway components, subcellular localization and changes in the level of expression of pathway constituents and regulators [82-83,95-98].

We have previously demonstrated that activated *HRAS*, *CRAF*, *AKT1*and mutated phosphatase-inactive *PTEN* genes confer drug resistance to breast cancer cells [2,3,5,99,100]. Cellular senescence is clearly a very important component in regulating cancer development and responding to DNA-damaging chemotherapeutics as well as anti-cancer dietary considerations [12,101-108]. However, it remains unclear as to how drug-resistant cells bypass the induction of cellular senescence.

In the following study, we examined the effects of activated PI3K/PTEN/Akt/mTOR and Ras/Raf/MEK/ERK pathways on the induction of cellular senescence in response to chemo-/hormonal therapy. Our results suggest that deregulation of PI3K/PTEN/Akt/mTOR, and to a lesser extent the Ras/Raf/MEK/ERK pathway, decreases drug-induced cellular senescence in response to chemo-/hormonal therapy.

## RESULTS

### Doxorubicin and Tamoxifen Induce Cellular Senescence in MCF-7 Breast Cancer Cells

**Figure 1:**
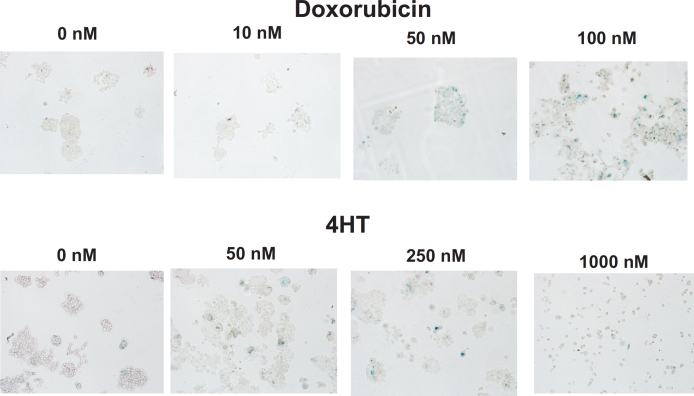
Effects of Doxorubicin and Tamoxifen (4HT) on the Induction of Cellular Senescence in MCF-7 Breast Cancer Cells Top Panels, MCF-7 cells were treated with increasing concentration of doxorubicin for 6 days and then stained for senescence associated (SA) β-galactosidase (SA-β-gal). Bottom Panels, MCF-7 cells were treated with increasing concentration of 4HT for 6 days and then stained for SA-β-gal.

**Figure 2:**
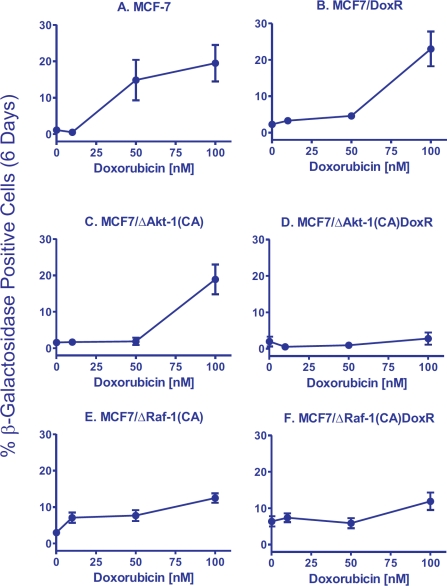
Effects of Activated Akt-1, Raf-1 and Selection for Doxorubicin-Resistance on the Induction of Cellular Senescence in Response to Different Concentrations of Doxorubicin in MCF-7 Derivatives Quantification of the induction of cellular senescence in response to doxorubicin treatment. 10 individual images were taken of different areas of the cover slip for each cell line, for a total of 1,000-3,000 cells per condition. The total number of SA-β-gal positive and negative cells was determined on each image, and the percentages of SA-β-gal positive from each culture condition and cell type were then determined and averaged.

We first examined the ability of doxorubicin and 4 hydroxy-tamoxifen (4HT) to induce senescence in p53 wild type (WT) and estrogen receptor (ER) positive MCF-7 breast cancer cells [2,3,5,99,100]. Cells were plated in 6 well plates containing an etched, gridded coverslip on the bottom of the well at 5 × 10^6^ cells/well. The MCF-7 cells adhered to the etched coverslips and essentially grew as colonies (Figure 1). After 6 days, the cells on the coverslips were processed for β-galactosidase staining as described [109]. Both doxorubicin (10-100 nM) and 4HT (50-1000 nM) induced senescence in MCF-7 cells in a dose-dependent fashion (Figure 1). At high concentrations of 4HT (1000 nM), MCF-7 cells did not grow and form colonies. Thus in subsequent experiments, lower doses of 4HT were used. Quantitation of the effects of doxorubicin and 4HT on MCF-7 cells is presented in Figures 2 and 4.

### Effects of Doxorubicin on the Induction of Cellular Senescence in Doxorubicin-Resistant MCF-7 Cells

We also isolated MCF-7 cells with increased resistance to chemotherapeutic drugs by culturing the cells in medium containing 25 nM doxorubicin. These cells were named MCF7/DoxR. The MCF7/DoxR cells were approximately 5.7-fold more resistant to doxorubicin than MCF-7 cells as determined by MTT analysis.

**Figure 3:**
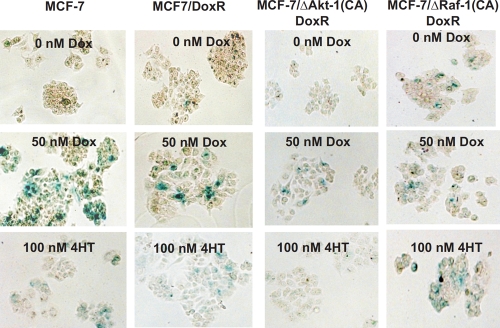
Effects of Doxorubicin and 4HT on the Induction of Cellular Senescence in MCF-7 Cells Containing Activated Akt-1, Raf-1 or Selected for Doxorubicin-Resistance Photomicrographs of wild type MCF-7 and doxorubicin-resistant ΔAkt-1(CA) or ΔRaf-1(CA) cells after staining for SA-β-gal. Top row: untreated cells, middle row: cells treated with 50 nM doxorubicin, bottom row: cells treated with 100 nM 4HT.

The ability of MCF-7 and MCF7/DoxR cells to undergo cellular senescence was quantified from β-galactosidase-positive cells in the presence of doxorubicin (10, 50 and 100nM) for 6 days (Figure 2, Panels A and B). While MCF7/DoxR cells displayed lower levels of senescence compared to MCF-7 cells at 10 and 50 nM, similar levels of senescence was achieved at 100 nM, suggesting that drug-resistance cells have a diminished ability to arrest (Figure 2, Panels A and B). Photomicrographs of the induction of cellular senescence in MCF-7 and the doxorubicin-resistant cells in response to doxorubicin treatment are presented in Figure 3.

### Ectopic Akt-1 Expression Promotes Resistance of MCF-7 Cells to Induction of Senescence after Doxorubicin Treatment

Since the PI3K/PTEN/Akt/mTOR pathway is frequently altered in breast cancer and associated with drug resistance, we used a retrovirus to stably-transfect MCF-7 cells with a constitutively-active Akt-1 gene [ ΔAkt-1(CA)]. Expression of activated Akt-1 alone was sufficient to increase the IC^50^ for doxorubicin by 2-fold in MCF7/ΔAkt-1(CA) [100]. Similar to MCF7/DoxR cells, expression of ΔAkt-1 reduced senescence 10-fold compared to MCF-7 cells when the cells were treated with 50 nM doxorubicin (Figure 2, Panels A and C).

We also isolated MCF7/ΔAkt-1(CA) cells with increased resistance to chemotherapeutic drugs by culturing the cells in medium containing 25 nM doxorubicin. These cells were named MCF7/ΔAkt-1(CA)DoxR. The MCF7/ΔAkt-1(CA)DoxR were 5-fold more frequently recovered from MCF7/ΔAkt-1(CA) than the MCF7/DoxR were from MCF-7 cell line. The MCF7/ΔAkt-1(CA)DoxR cells were approximately 6.7-fold more resistant to doxorubicin than control MCF-7 cells [100].

Interestingly, constitutive activation of Akt-1 in the drug resistant cells [MCF7/ ΔAkt-1(CA)DoxR] resulted in the complete abrogation of drug-induced senescence at all doses examined (Panel D), suggesting an interplay between active Akt-1 and other resistance mechanisms acquired by the MCF7/DoxR cells. We have observed that MCF7/DoxR cells overexpress activated ERK, but not activated Akt when cultured in medium containing doxorubicin. In addition, we have seen that MCF7/ΔAkt-1(CA) also overexpress activated ERK, suggesting the presence of both active pathways may serve to prevent drug-induced senescence.

### Effects of Ectopic Raf-1 Expression on the Induction of Senescence

Since we observed elevated activated ERK1/2 expression in MCF7/DoxR cells, the activity of the Raf/MEK/ERK cascade was manipulated in MCF-7 cells in order to determine whether signals transduced by this pathway control the induction of drug-induced senescence. MCF-7 cells were stably transfected with a constitutively-active Raf-1 gene [Raf-1(CA)] using a retrovirus [2,3]. Expression of constitutively-active Raf-1 increased resistance to doxorubicin by 9-fold compared to parental MCF-7 cells [2,3].

Consistent with prior reports, activated Raf-1 expression increased baseline cellular senescence in the absence of stress by 3-fold, indicating that MCF-7 cells were undergoing oncogene-induced senescence (OIS) (Figure 2, Panels A and E). MCF7/ΔRaf-1(CA) cells treated with 10 nM doxorubicin showed a 2.4-fold increase in cellular senescence, whereas similar treatments did not induce cellular senescence in either MCF-7 or MCF7/ΔAkt-1(CA) cells (Figure 2, Panels A, C and E). Interestingly, increasing concentrations of doxorubicin in MCF7/ΔRaf-1(CA) cells failed to induce senescence to as high levels as observed in MCF-7 or unselected MCF7/Akt-1(CA) cells.

We also isolated MCF7/ΔRaf-1(CA)DoxR cells with increased resistance to chemotherapeutic drugs by culturing the cells in medium containing 25 nM doxorubicin. These cells are approximately 5-fold more resistant to doxorubicin than MCF7/ΔRaf-1(CA) cells. The MCF7/ΔRaf-1(CA)DoxR cells had a 5.8-fold higher baseline level of cellular senescence than MCF-7 cells (Figure 2, Panels A and F) and a 2.1-fold higher baseline level of cellular senescence than MCF7/ΔRaf-1(CA) cells (Figure 2, Panels E & F). However, these MCF7/ΔRaf-1(CA)DoxR cells displayed a similar induction of senescence in response to doxorubicin treatment as MCF7/ΔRaf-1(CA) cells (Figure 2, Panels E & F).

### Active Akt-1 Prevents the Induction of Cellular Senescence Following Tamoxifen Treatment

**Figure 4:**
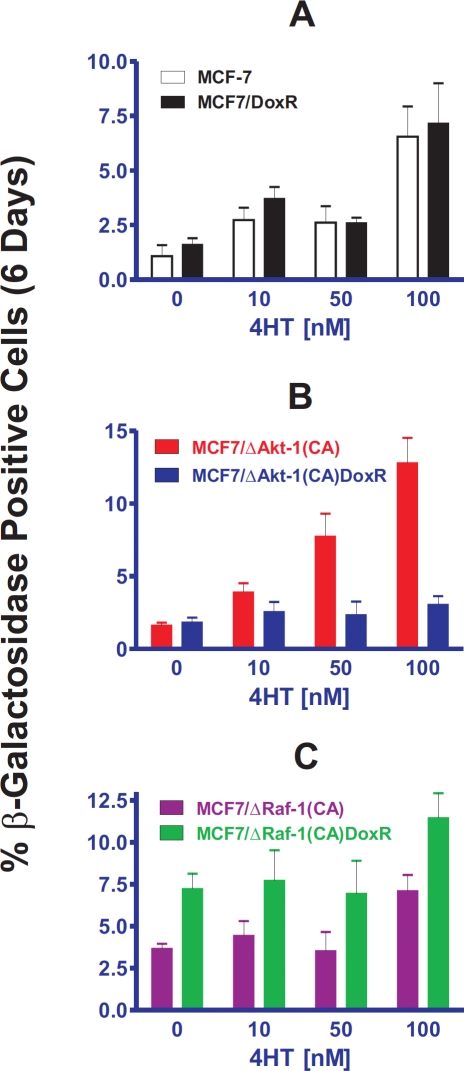
Effects of Activated Akt-1, Raf-1 and Selection for Doxorubicin-Resistance on the Induction of Cellular Senescence in Response to Different Concentrations of Tamoxifen in MCF-7 Derivatives Quantification of the induction of cellular senescence in response to 4HT treatment. 10 individual images were taken of different areas of the cover slip for each cell line, for a total of 1,000-3,000 cells per condition. The total number of SA-β-gal positive and negative cells was determined on each image, and the percentages of SA-β-gal positive cells from each culture condition and cell type were then determined and averaged.

Since chemotherapies are commonly administered in combination with hormonal therapy in ER-positive breast cancers, we sought to determine if drug resistance and oncogenic signaling through Akt-1 or Raf-1 effects induction of cellular senescence in response to 4-hydroxy tamoxifen (4HT). MCF-7 or MCF7/DoxR cells expressing constitutively-active Akt-1 or Raf-1 were treated with 10, 50 and 100 nM 4HT for six days then stained for β-galactosidase activity (Figure 4). Photomicrographs of the induction of cellular senescence in MCF-7 and the doxorubicin-resistant cells in response to 4HT or doxorubicin treatment are presented in Figure 3.

Similar levels of cellular senescence were observed in MCF-7 and MCF7/DoxR (Panel A) after 4HT treatment, suggesting that mechanisms of drug resistance are independent of senescence inducted by 4HT. Interestingly, ΔAkt-1(CA) expression did not prevent cellular senescence induction by 4HT treatment in the unselected MCF7/ΔAkt-1(CA) cells (Panel B), but potently suppressed senescence in doxorubicin-resistant MCF7/ΔAkt-1(CA)DoxR cells (Panel B). In contrast, Raf-1 activation in doxorubicin-resistant MCF7/ΔRaf-1(CA)DoxR cells did not suppress senescence by 4HT treatment (Panel C). Together, these results suggest that presence activated PI3K/PTEN/Akt/mTOR pathway in the presence of acquired drug resistant Raf/MEK/ERK pathway activation is sufficient to block drug-induced senescence to both doxorubicin and 4HT.

## DISCUSSION

In our studies, we determined the effects of the chemotherapeutic drug doxorubicin and the hormonal drug tamoxifen (4HT) on the induction of cellular senescence in MCF-7 and derivative cell lines which varied in their levels of activated Akt-1 or Raf-1 expression. Moreover, we also examined the induction of cellular senescence in cells which were selected for drug resistance by prolonged culture in medium containing 25 nM doxorubicin to determine whether drug resistance would alter the induction of senescence after either doxorubicin or 4HT treatment. An advantage of our investigation was that all cells had the same basic genetic background (MCF-7) and differed only in the levels of activated Akt-1 or Raf-1, or their resistance to doxorubicin.

A lower level of senescence was observed after 50 nM doxorubicin treatment of MCF7/DoxR than in MCF-7 cells. However, when the cells were treated with 100 nM doxorubicin, similar levels of cellular senescence were observed. These results demonstrated that the doxorubicin-resistant MCF7/DoxR wells were more resistant to the induction of senescence after doxorubicin treatment than unselected MCF-7 cells. However, the MCF7/DoxR doxorubicin-resistant phenotype did not prevent the induction of senescence in response to 4HT treatment. This indicates that acquired resistance to DNA damage-induced senescence does not protect against senescence induced by hormone blockade, and thus the two are likely to occur through independent signaling cascades.

While previous studies have shown that overexpression of either activated Akt-1 or Raf-1 results in resistance to doxorubicin [2,3,99,100], in our current work activated Akt-1 and Raf-1 expression have different effects on the induction of senescence after doxorubicin treatment. Overexpression of activated Akt-1 resulted in decreased induction of senescence compared with MCF-7 cells after 50 nM doxorubicin treatment. Drug-resistant MCF7/ΔAkt-1[CA]DoxR cells were even more resistant to the induction of senescence than either MCF7/ΔAkt-1[CA], MCF7/DoxR or MCF-7 cells (Figure 2). In contrast, a higher background level of cellular senescence was observed in untreated MCF7/ΔRaf-1(CA) and MCF7/ΔRaf-1(CA)DoxR cells compared to MCF-7 cells. However, the levels of cellular senescence observed after 100 nM doxorubicin treatment of MCF7/ΔRaf-1(CA) and MCF7/ΔRaf-1(CA)DoxR cells were not as high as observed in MCF-7 cells, suggesting that while there were higher levels of constitutive senescence in MCF7/ΔRaf-1(CA) and MCF7/ΔRaf-1(CA)DoxR cells, they were blocked in their ability to undergo as much senescence as MCF-7 cells.

MCF-7 cells contain WT p53 and doxorubicin induces p53 in MCF-7 cells [99]. Lower levels of p53 and downstream p21^Cip-1^were induced in doxorubicin-resistant MCF-7 cells which also contain activated Akt-1[99]. p53 plays key roles in the regulation of cellular senescence [38-40,43-46,110-120], DNA damage response [121-123], cell cycle progression [124-143], chromosome dynamics [144-146], apoptosis [147-151], sensitivity to chemotherapy [152-154], radiotherapy [107,155] tumorigenesis and metastasis [53,156-158], metabolism [159-161], cellular redox [41,162], hypoxia [61,75,136,163,164], autophagy [49,165,166] and aging [167] as well as other important biological processes. Often some of the purported functions of p53 are overlapping and even sometimes contradictory. p53 is regulated by other transcription factors [168,169], the proteins ATM [170], MDM2 and MDMX [174-180] and other interacting proteins [181-187] and miRNAs [171-173]. Small molecule inhibitors to proteins such as MDM2 which negatively regulate p53 have been developed [56,188-190]. Akt-1 can phosphorylate MDM-2 which can promote p53 destabilization. Decreased p53 and p21^Cip-1^levels may result in less cellular senescence in response to doxorubicin in the cells containing activated Akt-1 than in wild type MCF-7 cells.

The target genes of p53 and other members of the p53 family are clearly important in regulating many diverse processes [191-200]. p21^Cip1^ can activate pRb which can regulate entry into replicative senescence [12,201]. p53 also suppresses mTOR [38,40,44], however Akt can suppress the effects of p53 documenting the complexity of these pathways in how they regulate senescence induction [202-204]. miRNAs have also been shown to regulate critical genes involved in senescence such as pRb, p53, and PTEN [51,171-173,190,205]. Integrating these complex and often redundant interactions into a model by which p53 regulates cellular senescence in MCF-7 cells remains an ongoing process.

Recently it has been demonstrated by Blagosklonny and colleagues that the mTOR complex plays a critical role in regulating senescence and quiescence [30-32,38,40,206]. Inhibition of mTOR by rapamycin treatment in the presence of Nutlin-3A, will prevent the induction of senescence and result in p53-mediated quiescence [38, 40]. Interestingly, they also observed that inhibiting mTOR with rapamycin resulted in increased activation of “upstream” Akt [40]. This induction of Akt could have resulted from a shift in the equilibrium between mTORC1 and mTORC2, leading to higher levels of mTORC2 which resulted in Akt activation that prevented the induction of senescence by a mTORC1-independent mechanism [59].

Other studies by Blagosklonny's group demonstrated that knock down of TSC2, a negative upstream regulator of mTOR, could convert quiescent cells into senescent cells in the presence of Nutlin-3A [38]. Nutlin-3A is a MDM2 inhibitor, which results in p53 induction (accumulation). Inhibiting TSC2 resulted in increased mTORC1 activity which switched the cells from the quiescent into the senescent phenotype.

The effects of Akt on the induction of cellular senescence remain controversial. Often abnormal oncogene expression or tumor suppressor gene deletion (*PTEN*, *NF1* or *RB*) has been associated with “oncogene induced senescence” (OIS) which can be viewed as a protective mechanism to prevent tumor development [80,101]. However, recently it has been observed that Akt activation can suppress the senescence induced by mutant Ras signaling to promote tumorigenesis [207]. In this system, mutations at PI3K/Akt accelerated tumorigenesis potentially by bypassing the senescence induced by the activated Ras.

Activating mutations at oncogenes in the EGFR/Ras/PI3K/Akt pathway do not always result in OIS. *AKT1*, *FGFR3*, *PIK3CA*, *KRAS*, *HRAS*, and *EGFR* mutations have been found in some benign Seborrheic keratosis tumors which are not associated with increased cellular senescence. Interesting mutations in tumor suppressor genes *TSC1* and *PTEN* were not observed in this study [208].

Inhibition of Akt activity can also have effects resulting in the induction of senescence. Pharmacological suppression of Akt activity in combination with vitamin D3 can synergistically inhibit growth and result in the induction of senescence in prostate cancer cells [209]. This research demonstrates that quenching the effects of Akt can result in the induction of senescence. Expression of an inducible Akt gene suppressed temozolomide-induced Chk2 activation and G^2^ arrest and senescence in a glioma cell line model [210]. These studies indicate the protective effects of Akt with regards to certain chemotherapeutic drug treatments and support our findings that constitutive activation of Akt-1 can prevent the induction of senescence in MCF-7 cells after doxorubicin treatment.

Previously we have determined that there are higher levels of activated Akt-1 in the drug resistant cell line MCF7/ΔAkt-1ER*(Myr+)DoxR compared with MCF7/ΔAkt-1ER*(Myr+) cells which had not been selected for doxorubicin- or hormonal-resistance [99]. Growth in medium containing either doxorubicin or 4HT selects for ΔAkt-1(CA)-infected cells, as higher levels of Akt-1 expression provides a selective growth advantage in the presence of these drugs [99].

We have also shown that introduction of mutated forms of PTEN into MCF-7 cells which lacked lipid and protein phosphatase activity also suppressed the ability of doxorubicin to induce cellular senescence [5]. These cells which express the PTEN mutants were also more drug resistant than MCF-7 cells and also overexpressed activated Akt [5]. The PI3K/PTEN/Akt/mTOR and Raf/MEK/ERK pathways differed in their abilities to modulate the induction of cellular senescence in response to doxorubicin treatment in breast cancer cells as activated Akt-1 impeded senescence while activated Raf-1 did not, similar to the results of the present study.

Doxorubicin induces the Raf/MEK/ERK pathway in MCF-7 cells and higher levels of active ERK are detected in MCF7/DoxR cells than in drug sensitive MCF-7 cells. ERK can in some cases phosphorylate p53 (or additional proteins) and alter its activity. However, this increased ERK expression does not appear to prevent the induction of cellular senescence. In fact, a higher as a higher baseline level of cellular senescence was detected in MCF7/ΔRaf-1(CA) cells. Thus it can be argued that there is probably an additional modification(s) besides increased ERK expression in MCF7/DoxR which results in the decreased induction of senescence after doxorubicin treatment. While both MCF7/ΔAkt-1(CA) and MCF7/ΔRaf-1(CA) cells display increased resistance to doxorubicin compared with MCF-7 cells [2,3,100], they varied in terms of induction of senescence after doxorubicin treatment. Thus the genetic mechanisms responsible for chemotherapeutic drug resistance and senescence induction are different.

The ability of 4HT to induce cellular senescence in breast cancer is not well documented. 4HT likely induces reactive oxygen species (ROS) which in turn cause DNA damage and subsequently cellular senescence [211-213]. Similar levels of cellular senescence were observed in MCF-7 and MCF7/DoxR cells when they were cultured in medium containing 4HT. Thus the genetic modifications that occur in MCF7/DoxR cells which allow them to grow in medium containing 25 nM doxorubicin are not sufficient to prevent the induction of senescence induced by 4HT.

Induction of cellular senescence in response to 4HT differed in these models which expressed activated Akt-1(CA) or Raf-1(CA). In the MCF7/ΔAkt-1(CA) cells that had not been selected in doxorubicin, there was more cellular senescence than in the MCF-7 cells in response to 4HT treatment (compare in Figure 4 panels A and B). In contrast, with the MCF7/Akt-1(CA)DoxR cells that had been selected in doxorubicin, less cellular senescence in response to 4HT treatment was observed than in all the other cell lines examined. These results suggest that doxorubicin-selected drug resistant cells which overexpress activated Akt-1 are resistant to the induction of senescence upon 4HT as well as doxorubicin treatment. As stated previously, the doxorubicin-resistant cells express higher levels of activated Akt-1 than cells which have not been selected in doxorubicin. This higher level of Akt-1 confers increased resistance to 4HT [99,100].

Activated Raf-1 did not appear to suppress the induction of cellular senescence in response to 4HT treatment. Although in general, relatively constant levels of cellular senescence were observed in MCF7/ΔRaf-1(CA) and MCF7/ΔRaf-1(CA)DoxR cells in response to 4HT treatment as was seen after doxorubicin treatment of these cells. Taken together with the results observed with MCF7/DoxR cells, which express high levels of activated ERK, it is likely that increased levels of the Raf/MEK/ERK pathway does not inhibit the induction of cellular senescence induced by either 4HT or doxorubicin.

In some cases, the Ras/Raf/MEK/ERK pathway may induce cellular senescence [27,214-218]. Ras can activate both the Raf/MEK/ERK and PI3K/PTEN/Akt/mTOR pathways [6-8,59,60]. Other investigators have demonstrated that sustained Ras activation in endothelial cells can result in autonomous growth and senescence bypass and alter the differentiation status [219]. However, this altered phenotype was demonstrated to be regulated by the PI3K/Akt pathway.

These results have clinical significance as the PI3K/PTEN/Akt/mTOR pathway is often activated in breast cancer by mutations at *PIK3CA* and dysregulation of *PTEN*. Thus this pathway and downstream substrates such as the transcription factor Twist are critically involved in breast cancer and are targets for improved therapy [220-222]. Furthermore, drug resistance frequently develops in breast cancer after chemo- or hormonal-based therapies. Akt is frequently activated by upstream *PIK3CA* or *PTEN* mutations or gene silencing. *PTEN* can be mutated or silenced by various mechanisms in human cancer. Mutations occur which either delete the *PTEN* gene or alter its activity. These mutations which result in activated Akt expression may have effects on the induction of cellular senescence in response to chemotherapeutic drugs currently used to treat breast cancer patients. Our studies also indicate that just because a cell is drug resistant, that does not mean that it will be resistant to the induction of cellular senescence by a chemotherapeutic drug. Doxorubicin induced cell cycle arrest in the G^2^/M phase in both MCF7/ΔRaf-1 and MCF7/Akt-1 cells, yet these cells differed in the extent of cellular senescence after doxorubicin treatment. Clearly cell cycle regulatory proteins play key roles in regulation of cellular senescence [223]. Other studies have shown that certain proteins such as p21^Cip-1^are required in some cells for the induction of senescence but not for cell cycle arrest in response to HDAC inhibitors such as sodium butyrate [224]. Together these studies point to the complexities of drug resistance and cellular senescence.

## MATERIALS AND METHODS

### Cell Culture

MCF-7 cells were obtained from the American Type Culture Collection (ATCC) (Manassas, VA). Cell culture medium for MCF-7 cells consisted of Roswell Park Memorial Institute-1640 (RPMI 1640) medium (Invitrogen, Carlsbad, CA) supplemented with 10% (v/v) heat inactivated fetal bovine serum (FBS) as described [2].

### Transfection of MCF-7 cells with Akt-1 and Raf-1 Constructs

5 × 10^5^ MCF-7 cells were plated into 6-well cell culture plates (BD Biosciences, Mountainview, CA) and one day later infected with a retrovirus containing the various plasmid DNAs as described [3,5,99,100,109]. The nomenclature of the transfected cells is MCF7/ followed by the name of the introduced plasmid DNA. Stably transfected/infected cells were isolated by culture in medium containing 2 mg/ml G418 (Geneticin, Invitrogen).

### Cellular Senescence Assay

Senescent cells were identified by a senescence associated (SA) β-galactosidase (SA-β-gal) assay as described [109].
